# Remote web-based self-assessment of visual acuity versus ETDRS in patients with macular diseases: a method comparison study

**DOI:** 10.1186/s40942-025-00656-7

**Published:** 2025-03-14

**Authors:** Casper van der Zee, Leon Daniel Huang Huynh, Saskia Marijke Imhof, Jeannette Ossewaarde-van Norel, Redmer van Leeuwen, Robert Pieter Leendert Wisse

**Affiliations:** 1 Ophthalmology Department, University Medical Center Utrecht, Utrecht, the Netherlands; 2 Easee BV, Amsterdam, the Netherlands; 3 Heidelberglaan 100, Utrecht, 3584 CX the Netherlands

**Keywords:** eHealth, Macular diseases, Online eye testing, Ophthalmology, Remote eye care, Retina, Telemedicine, Telemonitoring, Visual acuity

## Abstract

**Background:**

Macular diseases (MD) lead to frequent clinic visits, involve time-consuming visual acuity (VA) measurements by professionals. Independent home measurements could improve efficiency. This study evaluates the agreement of a web-based test in MD compared to in-hospital measurements.

**Methods:**

Adults with MD were included at the University Medical Center Utrecht in March-July 2023. Users need a phone, computer, and 3m distance. The test uses Tumbling-E and triangles as optotypes. Primary outcome is the web-based vs. ETDRS Distance Visual Acuity (DVA). Secondary outcomes were test-retest variability (TRV), near visual acuity (NVA), and the Amsler grid. Outcomes were reported in mean differences and 95% Limits of Agreement (LoA).

**Results:**

89 eyes were included. The DVA mean difference was 0.03LogMAR(1.5 letters), SD0.17, LoA − 0.31;0.36LogMAR(-15.5;18 letters), TRV had a mean difference of 0.03(1.5 letters) SD0.14. The NVA mean difference was 0.13(6.5 letter) SD0.24, positive- and negative predictive values 0.93(95%CI = 0.82;0.98) and 0.71(95%CI = 0.51;0.86) respectively.

**Conclusions:**

The agreement of the DVA web-based test is on par with Snellen line assessment and subpar to ETDRS. We showed that elderly can perform this test independently at home, providing a time- and cost-saving opportunity. Developments should focus on the NVA since it can be a valuable adjunct to MD follow-up.

**Trial registration:**

the Dutch Medical Ethical committee (Medisch Ethische Toetsingscommissie; METC NedMec) registration number: 22–879/DB. Approved at 27-09-2022.

**Supplementary Information:**

The online version contains supplementary material available at 10.1186/s40942-025-00656-7.

## Background

Macular diseases (MD) are a significant cause of irreversible visual impairment, particularly among individuals over the age of 60 [1]. It is estimated that one in fifteen blind individuals is blind due to MD. The most predominant is age-related macular disease (AMD), which is predicted to increase from 196 to 288 million from 2020 to 2040 due to the growing and aging population [2]. Moreover, Diabetic macular edema (DME), a complication of diabetic retinopathy (DR), is a major cause of vision impairment, especially in working-age adults, with steady rising global prevalence [3]. Similarly, central serous chorioretinopathy (CSC) is a significant cause of central vision loss [4]. While CSC can sometimes resolve spontaneously, chronic or recurrent cases require ongoing observation to prevent long-term visual impairment. These MD have in common that frequent follow-ups for monitoring and treatment are deemed necessary to minimize preventable vision loss.

However, conventional in-office assessments require trained staff and specialized equipment. As demand for eye care increases due to a significant increase in prevalence of eye diseases, healthcare resources are becoming scarce [5]. Moreover, in-clinic visits pose a burden for patients such as travel time and expenses. This reduces patient satisfaction and adherence to follow-up of care [6]. In this context, telemedicine could aid to this stressed access to care.

This study assesses an online eye test (Easee), which is a web-based self-assessment of visual acuity. A major benefit of this digital test is its scalability; participants can independently perform the test if they can control a phone and a computer. Many online eye tests are available, either web-based or on iOS/android, but as yet only a minority are clinically validated and certified as a Medical Device [7, 8]. The validity of this remote distance visual acuity (DVA) testing has been reported previously in controlled settings [9, 10]. However the combination of DVA, near visual acuity (NVA), and Amsler grid was not researched yet and can be of particular relevance. To the same extent, we investigate whether a typical MD population is able to perform the digital test independently at home after experiencing the test in a supervised environment. This study reports on the agreement and usability of this test in MD patients.

## Methods

### Design

This prospective method comparison study was conducted at the University Medical Center Utrecht. All consecutive participants with MD from the outpatient academi clinic focused on MD were considered for participation from March-October 2023. A purposeful sampling method was used to prevent an overrepresentation of academic conditions, effectively meaning that the available daily slots for inclusion were allocated to patients with common diagnoses. Inclusion criteria were an established MD diagnosis by an ophthalmologist, age 18 or above, and adequate knowledge of the Dutch or English language. Exclusion criteria were VA-reducing comorbidities including but not limited to cataract, end-stage glaucoma, uveitis, deep visually impaired amblyopia, and a VA of 20/200 Snellen or worse which is the limit of the acuity range of the web-based test. Each patient received the in-hospital Early Treatment Diabetic Retinopathy Study (ETDRS) and the in-hospital web-based eye test on the same day in random order (further described as ‘conventional’ and ‘web-based’, respectively). This ETDRS vs. web-based comparison was the primary outcome. The web-based test was again assessed at home within 1–7 days. This web-based test at home was compared with the web-based in-clinic test, as a test-retest analysis which was the secondary outcome.

### Web-based test

**Fig. 1:**
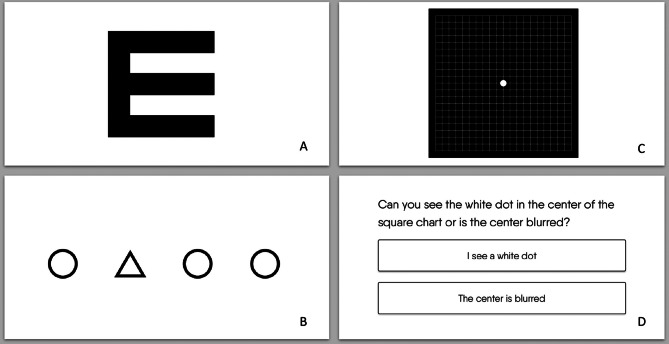
Charts presented during the digital test. (**A**) Tumbling E as presented on the phone and computer. (**B**) A proprietary optotype as presented on the phone and computer: triangles versus circles (users have to select which of the 4 symbols is different). (**C**) Amsler Grid presented on the computer (**D**) One of the three questions for the Amsler Grid presented on the computer

The web-based test was previously described in depth [11]. Summarized, this test is developed by Easee BV, an Amsterdam-based company, employing ISO13485 Quality Measurement System and is classified as Conformité Européenne (CE) class 2 A medical device according to the EU Medical Device Regulation 2017/745. The test is accessed via a website. Users need a phone, a computer, and 3 m of distance. The charts presented on the computer and phone during the web-based test are visualized in Fig. 1A-D. The phone functions as a remote. Participants were instructed to increase the brightness to the highest setting, sit at 3 m, and cover one eye with their hand or a paper. Participants are guided through the test with audio and visual instructions. The test order is randomized, and the test is programmed not to repeat the previous order of optotypes.

### Distance visual acuity

The primary outcome is DVA as measured by presenting VA, i.e., participants use their own spectacles. Both tests were performed at 3 m distance under the same lighting conditions. The conventional test used the backlight luminance protocol to measure Logarithm of the Minimum Angle of Resolution (LogMAR) on the Early Treatment of Diabetic Retinopathy Study (ETDRS) chart [12]. This chart consists of five letters per row and was measured per correct letter. The ETDRS is considered the gold standard for measuring VA in MD because due to precision and reliability [13–15]. Each patient was asked to identify the first letter on the side of each line until a letter was missed. The patient was then asked to identify each letter from the row above until the correctly identifing all letters in a single row. They continued to identify letters until the missing three letters on one line. Then, participants were asked to try the line underneath once. The single-letter method was used, meaning each correctly identified letter was counted. The remote test used tumbling-E’s and triangles (Fig. 1A). The setup was prepared in advance. No direct help was offered during the test, but assistance by a spouse was allowed, resembling the home situation.

### Near visual acuity

The conventional NVA was measured per eye with presbyopic correction in standard office illumination (> 500 lx) to the ETDRS near vision chart (#729000) according to protocol [16]. The chart was held at 40 cm, and each patient was asked to identify the first letter on the left side of each line, beginning with the 20/400 line on the left side, until a letter was missed. The patient was then asked to identify each letter from the row above until the patient correctly identified all five letters on a single row. They continued to identify letters until the patient missed three letters on one line. At that point, participants were asked once to try the line underneath. The single-letter method was used, meaning each correctly identified letter was counted. The near web-based test was performed under the same lighting conditions and distance as the conventional NVA. Participants were tested using Tumbling-E’s and triangle charts on the computer.

### Amsler grid

The Amsler grid iscommonly used at home by assessing central visual field for metamorphopsia (visual distortions) and scotomata (partial loss). Participants answer the following questions when focusing on a white dot: “*Do you see the white dot*,* or is it blurry*? *Are the lines bent or straight? Are any of the boxes missing*” When the answer was ‘blurry’, ‘bent’, or ‘yes’, the test was interpreted as positive. The conventional chart is a white-on-black 10 × 10 cm card, also known as the *Yannuzzi card*. It is preferred to black-on-white when VA is below 20/50. [17]Each eye is tested separately at 33 cm in standard office illumination using presbyopic correction. The web-based test is tested in the same conditions, visualized on the computer screen (Fig. 1C and D).

### Test-retest variability

Participants were requested to perform the same unsupervised DVA web-based retest at home within 7 days after consultation. The Test-Retest Variability (TRV) is compared to the in-clinic web-based DVA test. Participants receiving intravitreal injections (IVI) were requested to perform the retest on day six or seven.This is because IVIs are known to temporarily cause blurred vision, while a gain in VA due to treatment is limited within the first week. No instructions were given regarding lighting at home. IVI’s were only administered after testing visual acuity, not before. Only IVIs in a treat-and-extend regime were administered, no initial IVIs as a loading dose were provided.

### Power

This study is powered on the primary outcome, DVA. The 95% Limits of Agreement (LoA) of > 0.15LogMAR (7.5 letters) with a mean difference of 0.03 LogMAR is considered relevant [20, 21], with a standard deviation (SD) of 0.06 LogMAR. We assumed an α of 0.05 and a power of 90%. With a paired two-tailed t-test, 44 measurements are required (88 eyes).

### Statistical analysis

The following data were collected as baseline parameters: sex, age, and inclusion of one or both eyes. Additionally, the DVA web-based test time for two eyes was reported for the in-clinic measurement, including the set-up phase of the test. As the test time includes the set-up phase, the time was only analyzed in participants which included two eyes in the study. Results are reported in mean differences with 95% confidence intervals (CI), SD, and the LoA, visualized in Bland-Altman (BA) plots [22]. The BA analysis is commonly used to quantify the agreement between two methods of the same variable in. Correlation- and regression analyses are not recommended in BA analysis, and therefore not reported [23]. Subgroup analyses were perfomed on eyes with an absolute difference > 0.15LogMAR (7.5 letters). For the Amsler grid, we calculated the sensitivity, specificity, and positive- and negative predictive values using the physical Amsler chart as a reference.

Differences were tested using a two-sided paired sample t-test, a chi-square test, or a Fischer exact test. Results were considered significant when *p* < 0.05 corrected for multiplicity using Bonferroni correction and reporting adhered to the CONSORT and STROBE guidelines [24, 25]. The data were assessed for normality. A multivariable Generalized Estimating Equation (GEE) analysis is performed to correct for the inclusion of both eyes, bilaterality, age, sex, and MD. Missing cases were neither included, nor imputed.

### Ethical considerations

The study was performed in accordance with local laws and regulations, the Declaration of Helsinki, and the 2015 Standards for Reporting Diagnostic Accuracy Studies (STARD) [26]. The data was stored on *General Data Protection Regulation* (GDPR) and *Health Insurance Portability and Accountability Act* (HIPAA) compliant servers in the EU. Medical-ethical communication was filed to the Medisch Ethische Toetsingscommissie (METC NedMec) under reference 22–879/DB and Approved at 27-09-2022. All included participants were registered with informed consent.

## Results

**Fig. 2:**
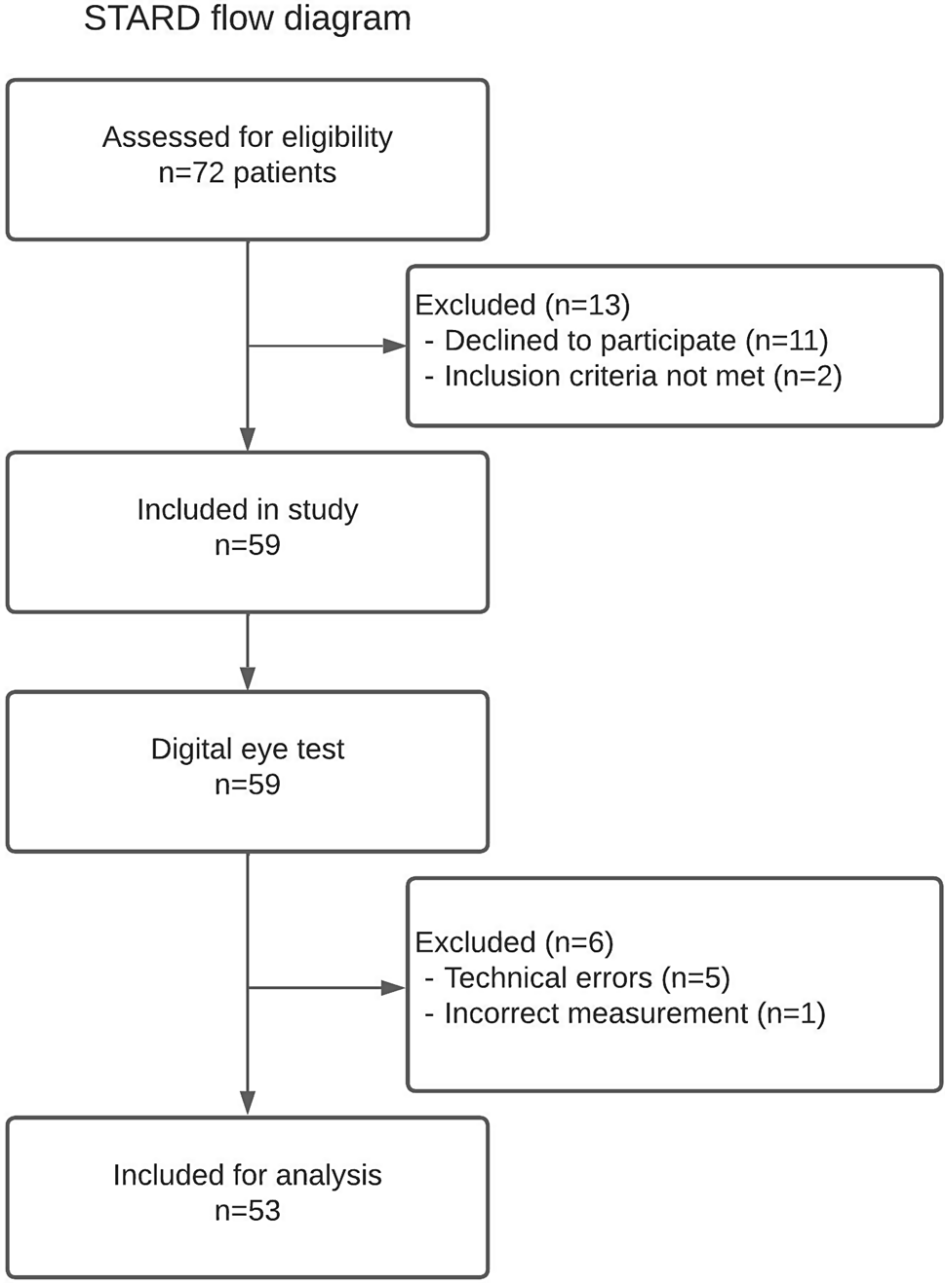
STARD flow diagram illustrating inclusion flow of participants (n) with macular diseases. All included participants underwent the digital (index test) and conventional assessments (reference test) of distance visual acuity

89 eyes from 53 participants were included (Fig. 2). The clinical characteristics are summarized in Table 1. The mean age is 70.7 years (range 36–93). The majority was female (58.5%) and consisted of AMD participants (65.2%). Mean DVA is 0.23LogMAR SD0.21 (20/34 Snellen). VA was missing in six eyes due to technical errors (data was not saved), and one patient was excluded due to a DVA lower than 20/200 Snellen. In one patient, the ETDRS was incorrectly administered. No adverse events or complications were recorded.

**Table 1:** Clinical characteristics of the study population. DVA: presenting distance visual acuity; LogMAR: logarithm of the minimum angle of resolution; CNV: choroid neovascularization;. *Other macular diseases include diabetic retinopathy, Irvine Gass, hemi-retinal vein occlusions

Clinical characteristics		Values
**N in patients **(*n*=53)		
Age (years), mean (SD)		70.7 (11.3)
Age (years), range		36– 93
Sex, n (%)	Female	31 (58.5)
Patients with bilateral macular disease, n (%)		36 (67.9)
Eye, n (%)	OD	42 (47.2)
Total test duration in clinic for both eyes (minutes), mean (SD)**		17.7 (4.4)
**N in eyes **(*n*=89)		
DVA conventional assessment (LogMAR), mean (SD)		0.23(0.21)
DVA conventional assessment (Snellen)		20/34
NVA conventional assessment (LogMAR), mean (SD)		0.37(0.26)
NVA conventional assessment (Snellen)		20/47
Macular Diseases, n (%)	Late-stage AMD	47 (52.8)
	Early-stage AMD	11 (12.4)
	Central Serous Chorioretinopathy with CNV	14 (15.7)
	Pseudoxanthoma Elasticum with CNV	8 (9.0)
	Other Macular Diseases*	9 (10.1)

### DVA accuracy: ETDRS vs. in-clinic web-based test

**Fig. 3:**
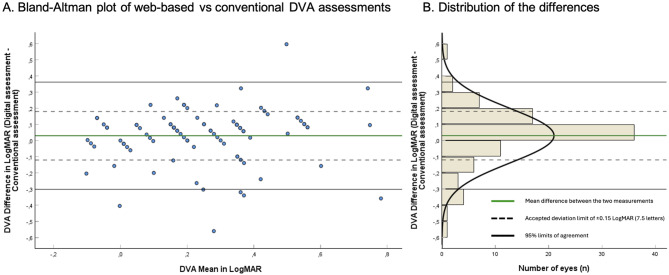
(**A**) Bland-Altman plot reporting the difference of the presenting distance visual acuity (DVA) between the web-based test and the conventional ETDRS test. *N* = 89 eyes. The y-axis represents the differences between both measurements of one eye, the x-axis the mean of these measurements. Each circle represents one eye. The black linkes constitute the 95% Limits of Agreement: -0.31 to 0.36 LogMAR, or -15.5 to 18 letters. (**B**) Distribution histogram summarizing the data shown in panel A. DVA: Distance visual acuity. LogMAR: logarithm of the minimum angle of resolution. 0.15 LogMAR = 7.5 letters

The mean difference between both tests was 0.03LogMAR (1.5 letters) Confidence Interval (CI)= -0.01;0.07, SD0.17. The BA-plot is reported in Fig. 3. The LoA ranged − 0.31 to 0.36 LogMAR (-15.5 to 18 letters), with no indication of proportional bias. 64/89 eyes (71.9%) fell within ± 0.15LogMAR (± 7.5 letters). The average web-based test time for both eyes was 17.7 min SD4.4.

### Test-Retest variability: web-based vs. web-based re-test

**Fig. 4:**
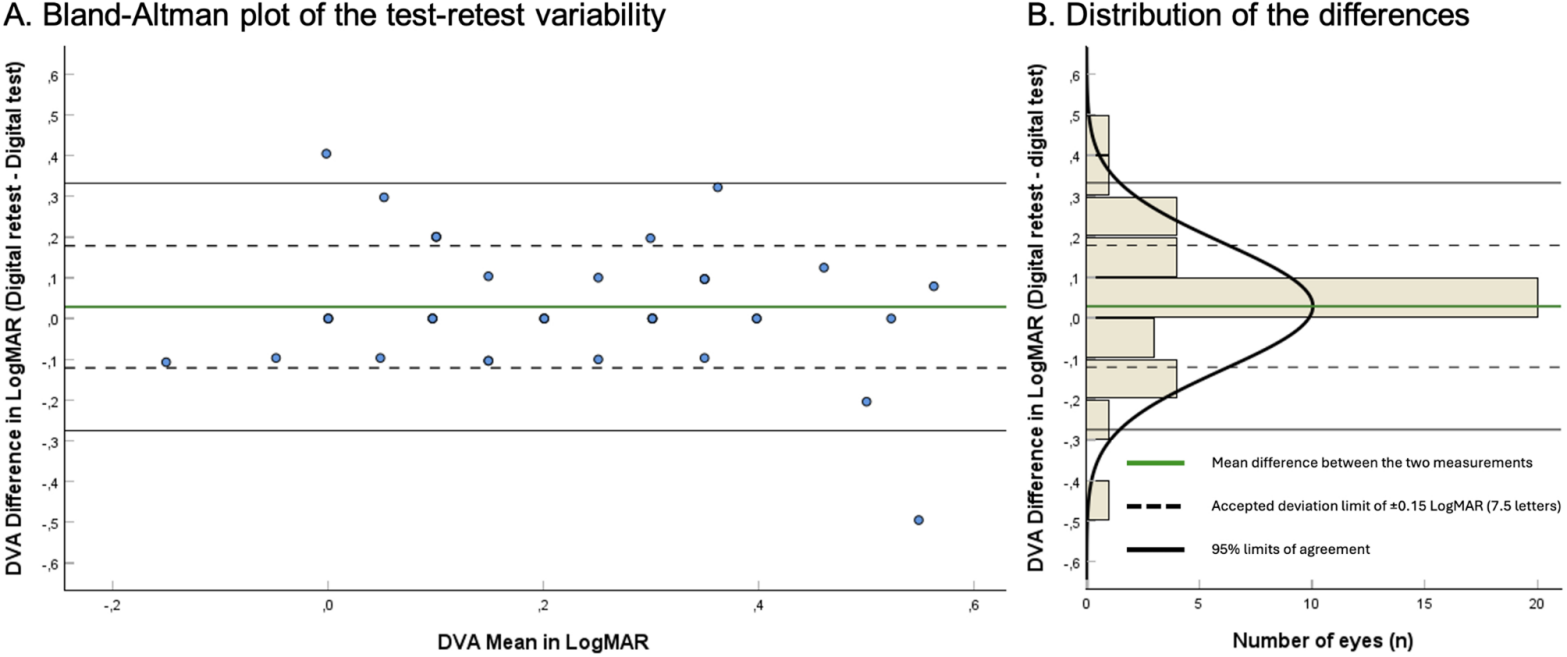
(**A**) Bland-Altman plot reporting the test-retest difference of the distance visual acuity (DVA) between the web-based test and the conventional ETDRS test. *N* = 39 eyes. The y-axis represents the differences between both measurements of one eye, the x-axis the mean of these measurements. Each circle represents one eye. The black linkes constitute the 95% Limits of Agreement: -0.28 to 0.33 LogMAR, or -14 to 16.5 letters. (**B**) Distribution histogram summarizing the data shown in panel A. DVA: Distance visual acuity. LogMAR: logarithm of the minimum angle of resolution. 0.15 LogMAR = 7.5 letters

Twenty-three participants (*n* = 39 eyes) performed the retest (mean interval 5.3 days, SD2.5). The mean age between the patient that did and did not perform the web-bases retest was not statistically different (68 ± 9.4 vs. 72.7 ± 12.3, *p* = 0.131). The mean DVA difference was 0.03LogMAR (1.5 letters) CI=-0.02;0.08, SD0.15, and the LoA ranged − 0.28 to 0.33 (-14 to 16.5 letters). The BA-plot is presented in Fig. 4.

### Subgroup: DVA ETDRS vs. in-clinic web-based test

The subgroup analysis performed on poor performers (difference between tests > 0.15 *n* = 25 eyes, 7.5 letters) compared to good performers (difference ≤ 0.15 LogMAR, *n* = 64 eyes) is reported in Table 2. Participants with lower VA had a higher variance (0.20 vs. 0.31LogMAR, 20/32 vs. 20/41 Snellen, *p* = 0.029), as expected. There was an age-related trend indicating elderly exhibited more variance, yet not statistically significant (*p* = 0.057).

**Table 2:** Subgroup analysis of distance visual acuity (DVA) ≤0.15 vs. >0.15 LogMAR of the digital test LogMAR: logarithm of the minimum angle of resolution. CSR: central serous retinopathy, PXE: Pseudoxanthoma elasticum. *Other macular diseases include diabetic retinopathy, hemi-central retinal vein occlusion due to hypertensive retinopathy and hemi-retinal vein occlusion [1]. Measured by the conventional Amsler chart

		Online test performance LogMAR ≤0.15 *N* = 64 eyes	Online test performance LogMAR >0.15 *N* = 25 eyes	*p*-values
Age (years), Mean (SD)		68.8 (11.9)	75.4 (8.2)	0.057
Female, n (%)		22 (57.9)	9 (60.0)	0.889
Conventional DVA, LogMAR, mean (SD) Snellen		0.20 (0.19) 20/32	0.31 (0.24) 20/41	0.029
Eyes with signs of scotoma^1^, n (%)		31 (50.0)	10 (45.5)	0.714
Eyes with signs of metamorphopsia^1^, n (%)		35 (56.5)	10 (45.5)	0.374
Macular Diseases, n (%)	Late-stage AMD	35 (54.7)	12 (48.0)	0.823
	Early-stage AMD	7 (10.9)	4 (16.0)	
	CSR with CNV	11 (17.2)	3 (12.0)	
	PXE with CNV	5 (7.8)	3 (12.0)	
	Other*	6 (9.4)	3 (12.0)	

### NVA accuracy: ETDRS vs. in-clinic web-based test

**Fig. 5:**
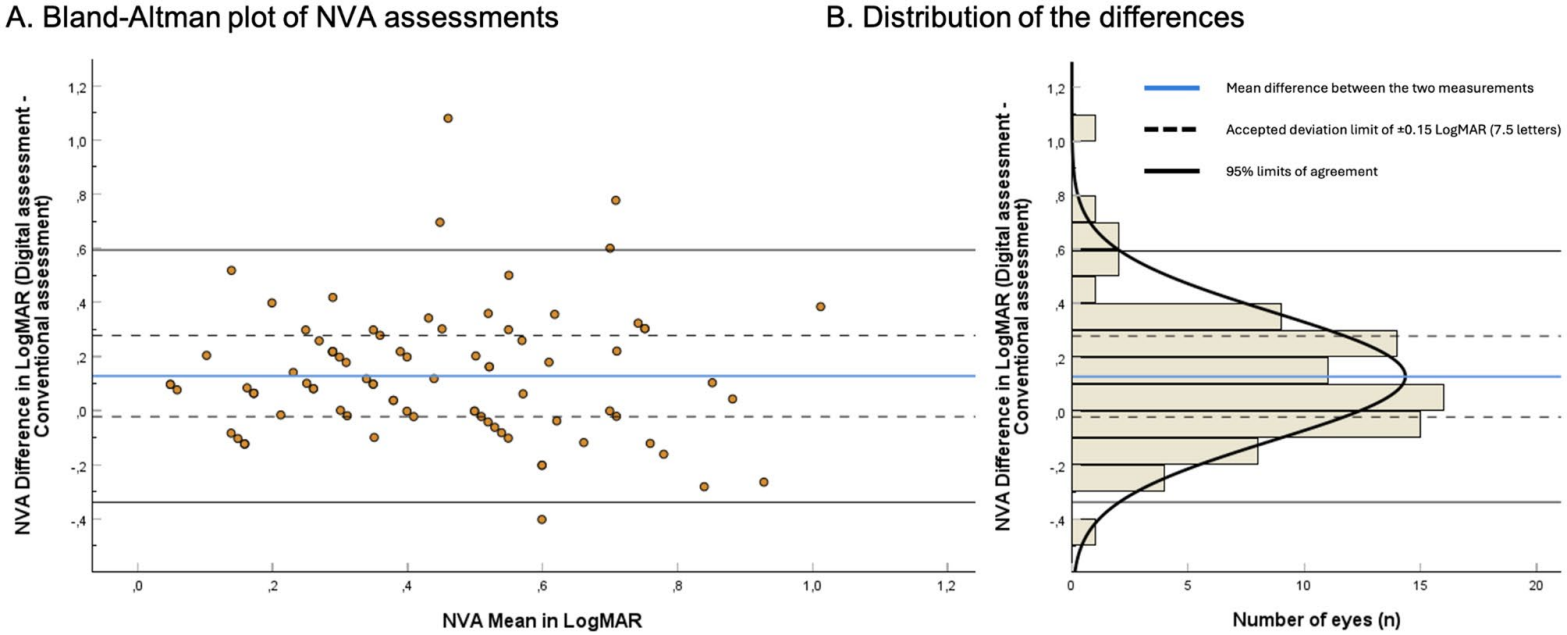
(**A**) Bland-Altman plot reporting the near visual acuity (NVA) differences between the web-based test and the conventional ETDRS test. *N* = 85 eyes. The y-axis represents the differences between both measurements of one eye, the x-axis the mean of these measurements. Each circle represents one eye. 95% Limits of Agreement: -0.34 to 0.59 LogMAR, or -17 to 29.5 letters. (**B**) Distribution histogram summarizing the data shown in panel A. DVA: Distance visual acuity. LogMAR: logarithm of the minimum angle of resolution. 0.15 LogMAR = 7.5 letters Declerations

Fifty-two participants (*n* = 85 eyes) performed the web-based test. The mean difference was 0.13LogMAR (6.5 letters) SD0.24, CI = 0.07;0.18 and the LoA ranged − 0.34 to 0.59LogMAR (-17 to 29.5 letters). The BA-plot is presented in Fig. 5. Low NVA was underestimated by the web-based test.

### Subgroup: NVA: ETDRS vs. in-clinic web-based test

The subgroup analysis performed on poor performers (difference between tests > 0.15, *n* = 39 eyes, 7.5 letters) vs. good performers (≤ 0.15 LogMAR, *n* = 46 eyes) is reported in Table 3. Again, participants with worse VA report a higher variance (0.32 vs. 0.44LogMAR, 20/42 vs. 20/55, *p* = 0.041).

**Table 3:** Subgroup analysis of near visual acuity (NVA) ≤0.15 vs. >0.15 LogMAR of the digital test LogMAR: logarithm of the minimum angle of resolution. CSR: central serous retinopathy, PXE: Pseudoxanthoma elasticum. *Other macular diseases include diabetic retinopathy, hemi-central retinal vein occlusion due to hypertensive retinopathy and hemi-retinal vein occlusion [1]. Measured by the conventional Amsler chart

		Online test performance LogMAR ≤0.15 *N* = 46 eyes	Online test performance LogMAR >0.15 *N* = 39 eyes	*p*-values
Age (years), Mean (SD)		70.7 (11.2)	71.0 (9.5)	0.903
Female, n (%)		26 (56.5)	22 (56.4)	0.992
Conventional NVA, LogMAR, mean (SD) Snellen		0.32 (0.22) 20/42	0.44 (0.29) 20/55	0.041
Eyes with signs of scotoma^1^, n (%)		19 (43.2)	22 (56.4)	0.229
Eyes with signs of metamorphopsia^1^, n (%)		26 (59.1)	19 (48.7)	0.344
Macular Diseases, n (%)	Late-stage AMD	27 (58.7)	16 (41.0)	0.077
	Early-stage AMD	4 (8.7)	7 (17.9)	
	CSR with CNV	4 (8.7)	10 (25.6)	
	PXE with CNV	4 (8.7)	4 (10.0)	
	Other*	7 (15.2)	2 (5.1)	

### Amsler grid

Fifty-one participants performed the web-based Amsler test (*n* = 84 eyes). The sensitivity and specificity are reported in Table 4. Positive and negative predictive values are 0.93 (CI = 0.82;0.98) and 0.71 (CI = 0.51;0.86), respectively (supplementary Table 3a-c. The subgroup analysis (Table 5) reports that participants with a positive Amsler have a decreased VA compared to a negative Amsler (*p* = 0.024 and *p* = 0.020, respectively). No difference is reported in ≤ or > 0.15LogMAR.

**Table 4:** Sensitivity and specificity of the digital Amsler grid compared to the physical Amster chart. TP: true positive, FP: false positive, FN: false negative, TN: true negative. TP = true positive, FP = false positive, FN = false negative, TN = true negative

Eyes^1^	TP^1^	FP^1^	FN^1^	TN^1^	Sensitivity^2^	Specificity^2^
84	52 (61.9%)	4 (4.8%)	8 (12.5%)	20 (23.8%)	86.7% (0.75-0.94)	83.3% (0.62-0.95)

^1^ In n (%)

^2^ In % (95% Confidence Interval)

**Table 5:** Subgroup analysis of the conventional Amsler positive vs. negative score of the digital test

	Negative Conventional Amsler *N* = 60 eyes	Positive Conventional Amsler *N* = 24 eyes	*p*-values
Age (years), Mean (SD)	69.8 (10.8)	72.3 (8.0)	0.309
Female, n (%)	37 (61.7)	10 (41.7)	0.095
Conventional DVA, LogMAR, mean (SD) Snellen	0.25 (0.20) 20/36	0.15 (0.19) 20/28	0.024
Conventional NVA, LogMAR, mean (SD) Snellen	0.42 (0.26) 20/53	0.27 (0.22) 20/37	0.020
Digital DVA, n (%) > 0.15 LogMAR (7.5 letters)	14 (23.3)	8 (33.3)	0.346
Digital NVA, n (%) > 0.15 LogMAR (7.5 letters)	28 (46.7)	11 (47.8)	0.925

### Generalized estimating equations

The GEE-analysis revealed no significant association between VA and any of the parameters (Supplementary Table 1). Also, baseline variables (age, sex, VA or bilaterality) did not impact the poor-VA subgroup analysis (i.e., > 0.15LogMAR, 7.5 letters), indicating that age and the severity of MD were interrelated (Supplementary Table 2).

## Discussion

The aim was to investigate the agreement and usability of a web-based VA test in MD patients compared to the ETDRS chart. We found a negligible mean difference of 0.03LogMAR (1.5 letters), indicating that the digital test does not systematically over- or underestimate DVA. The LoA ranged − 0.31 to 0.36LogMAR (-15.5 to 18 letters). In comparison, studies report variabilities ranging ± 0.14 up to ± 0.18 in ETDRS (± 7 and ± 9 letters) and ± 0.18 up to ± 0.24 in Snellen single letter testing (± 7 and ± 12 letters) [13, 14]. Using the line assignment method, the variability of Snellen is ± 0.33LogMAR (± 16.5 letters) [14, 15]. 

72% of measurements were within clinically relevant deviation (± 0.15LogMAR, 7.5 letters). All participants (mean age 71, range 36–93, mean DVA 20/32 Snellen) were able to complete the digital test and reported comparable outcomes at home (mean difference 0.03LogMAR or 1.5 letters, LoA − 0.28 to 0.33 LogMAR, -14 to 16.5 letters). Uncontrolled conditions at home did not affect results. Thus, following in-clinic exposure, at-home testing appeared to be feasible in this elderly population.

Three phenomena should be taken into consideration when interpreting these results. First, measuring repeated VA always exhibits variability, e.g. due to fatigue. Second, variability is inevitable when comparing different optotypes and charts, e.g. due to the conversion effect and a difference in crowding [27]. Third, variability typically increases in subjects with poorer VA, also observed in this study [15]. 

The NVA SD is ± 0.24 (12 letters). This is higher than expected, as literature reports SD to be ± 0.12 and ± 0.19 LogMAR (6 and 9.5 letters) [28, 29]. Notably, our population had a sub-par VA while literature assessed healthy individuals. When VA approaches ≤ 0.5 Snellen (20/40 Snellen, over half our population), NVA was observed in prior research to decrease abruptly, which increases the variability [30]. This is a focus for development since studies suggest that NVA is relevant for assessing therapy efficacy [31]. 

The sensitivity and specificity of the digital Amsler compared to conventional were 87% and 83%, respectively [32]. The Amsler itself is not a precise tool. However, due to its practicality and low costs, it is commonly used. With the current false negatives and false positives, the tool is suitable for screening. However, the false negatives in particular (12.5%) should be assessed and improved in a future study.

The primary aim was to investigate whether this remote test was reproducible and feasible for a broad group of MD rather than to restrict to one particular diagnosis. This improved generalizability. This population, often of age, bring additional challenges such as having retinal symptoms (e.g. metamorphopsia or scotoma’s), being a suspect for digital illiteracy, and having decreased visual acuity. Future studies should address repeatability (variation under identical conditions), and the within-diagnose variability.

### Strengths and limitations

A strength is the inclusion of a representative elderly MD population. Telemedicine is responding to the increasing demand for healthcare. However, elderly are excluded under the false assumption that telemedicine is unsuitable due to limited digital literacy [33]. Our study shows all elderly completed the web-based test, in line with previous research [9]. Also, we conducted the TRV to analyze the unsupervised setting. This study also has limitations. First, only participants who were somewhat interested in telemedicine participated. However, we proved that all were capable to independently perform the web-based VA test and it serves no purpose researching a population without digital skills. In the Netherlands, 86% of people aged 65–75 years are in possession of a smartphone with internet and we assume that this percentages increases with the coming generation [34]. Second, we acknowledge that a training effect could occur as patients are familiar with VA testing. Patients might memorize optotypes and decreased crowding effect over time. For the in-clinic web-based test vs. ETDRS, test order is randomized, reducing this effect. For the test-retest, a learning effect is presumed. This retest is taken unsupervised in a home environment and performed 1–7 days later, with the test programmed not to repeat the previous order. These outcomes are not materially different when we consider a compound effect of a comparable systematical bias of 0.03 LogMAR (1.5 letters; meaning the test is robust), a more challenging test environment (without supervision and doing the setup independently), and the aforementioned potential learning effect (off-setting the effects of the unsupervised environment). We demonstrate that with a one-time supervised training, the home performance of the test is comparable to the clinical setting. Third, the prophylactic IVIs might have affected the results. To our knowledge, no previous studies have reported about VA increase gains one week after anti-VEGF injections. Therefore, we consider the short-term effects of anti-VEGFs to be negligible. Fourth, this study assesses various types of MD and does not account for each population’s needs separately. The primary aim was to investigate whether this test was feasible for a broad group of patients with MD. Many eye diseases can lead to macular function problems, and we deliberately aimed to include patients based on their likelihood of exhibiting functional macular problems rather than restrict our research to one diagnosis. This population brings additional challenges increasing the variability and generalizability, as previously mentioned. However, the within-disease variability remains a knowledge gap that should be addressed in future research.

Moreover, it should be assessed in the future whether the results of this academic population can be extrapolated to routine practice clinics in an implementation study. Last, we measured the ETDRS chart from the line where five letters were identified correctly down the chart, instead of identifying every letter top-down. The latter method gives additional information on the presence of scotomata. This effect could not be quantified in this study, though was mitigated by including the Amsler results.

### Comparison with prior work

More studies evaluated the accuracy of this web-based test [9, 11]. Notably, the distribution of differences was higher in this study. However, participants were older and had considerably lower VA. This study was the first to assess an Amsler Grid. Many other tools for are available, though most are not validated and often tested on healthy individuals or non-elderly [7]. Those that are, were compared in strictly controlled settings [35]. One of these tests is the OdySight app, measuring VA and Amsler in macular degeneration. They assessed Tumbling-E and ETDRS and reported comparable LoAs (-0.30 to 0.24 LogMAR, -15 to 12 letters) [36]. Yet, participants were younger (mean age 64 vs. 71). Another study reports the myVisionTrackx app, assessing tumbling-E and Landolt-C charts compared to ETDRS in maculopathy. Results are comparable to this study, yet the authors do not measure NVA or the Amsler Grid (mean difference − 0.07 or 3.5 letters, LoA ± 0.35LogMAR or ± 17.5 letters) [37]. 

On a side note, the follow-up of MD is based on Optical Coherence Tomography (OCT). It is important to underline that VA measurements is no replacement for the OCT.

## Conclusions

We report that this web-based VA test is on par with the conventional Snellen DVA line assessment and subpar to the ETDRS chart. We showed that elderly with basal digital proficiency can perform this remote test at home, providing interesting opportunities considering time- and cost savings. Developments should focus on improving the NVA, since it it can be a valuable adjunct to follow up MD.

## Electronic supplementary material

Below is the link to the electronic supplementary material.

Supplementary Material 1

## Data Availability

The dataset analysed during the current study is available from the corresponding author upon reasonable request.

## Abbreviations

GEE: Generalized Estimating Equation
LogMAR: Logarithm of the Minimum Angle of Resolution
DVA: Distance Visual Acuity
NVA: Near Visual Acuity
ETDRS: Early Treatment Diabetic Retinopathy Study
ODS: Oculus Dexter Sinister (right and left eye)
STARD: Standards for Reporting Diagnostic Accuracy Studies
IVI: Intravitreal injections
UMCU: University Medical Center Utrecht
LoA: Limits of Agreement
CI: 95% Confidence Interval
AMD: Age-related Macular Degeneration
CNV: Choroidal Neovascularization
CSC: Central Serous Chorioretinopathy
PXE: Pseudoxanthoma Elasticum
TRV: Test-Retest Variability
BA: Bland-Altman
