# Supplementary material for: Remote web-based self-assessment of visual acuity versus ETDRS in patients with macular diseases: a method comparison study
Source: Int J Retina Vitreous. 2025 Mar 14;11:29. doi: 10.1186/s40942-025-00656-7 (PMC11907903; doi:10.1186/s40942-025-00656-7)
Supplement: Supplementary file 1 — Supplementary Material 1 [file 40942_2025_656_MOESM1_ESM.pdf]

Additional file 1: Supplementary tables 1-3ac

Parameter	B	SE	95% Confidence Interval Lower	95% Confidence Interval Upper	Hypothesis Test Chi-Square	Hypothesis Test df	Hypothesis Test Sig.
(Intercept)	,114	,1121	-,106	,334	1,037	1	,308
Sex	-,148	,1878	-,516	,220	,621	1	,431
Age (years)	-,001	,0015	-,004	,002	,511	1	,475
Sex* Age (years)	,002	,0028	-,004	,007	,424	1	,515
(Scale)	,029						

Supplementary Table 1. GEE Analysis for bilaterality
Dependent Variable: DVA Difference. Model: (Intercept), Sex, Age (years), Sex * Age (years)

	OD (N = 42 eyes)	OS (N = 47 eyes)	*p*-values
Conventional DVA in LogMAR, mean (SD)<br>Snellen	0.23 (0.24)<br>20/34	0.23 (0.19)<br>20/34	0.984
Conventional NVA in LogMAR, mean (SD)<br>Snellen	0.36 (0.25)<br>20/46	0.39 (0.27)<br>20/49	0.654

Supplementary Table 2. Subgroup analysis right versus left eye (OD and OS).

		Conventional Amsler Positive N	Positive %	Negative N	Negative %	Total N	Total %
Digital Amsler	Positive	52	86.7	4	16.7	56	66.7
	Negative	8	13.3	20	83.3	28	33.3
Total		60		24		84	100

Supplementary Table 3a. Crosstable comparing the conventional (paper) Amsler grid with the Digital Amsler Grid. "Positive" means the patient answered either or both 'blurry', 'bent', or 'yes' to the three Amsler questions respectively.

		Conventional Amsler Positive N	Positive %	Negative N	Negative %	Total N	Total %
Digital Amsler	Positive	31	75.6	3	7.0	34	40.5
	Negative	10	24.4	40	93.0	50	59.5
Total		41		43		84	100.0

Supplementary Table 3b. Crosstable comparing the conventional (paper) Amsler grid with the Digital Amsler Grid. "Positive" means the patient saw bent lines on the Amsler grid (question 2).

		Conventional Amsler Positive N	Positive %	Negative N	Negative %	Total N	Total %
Digital Amsler	Positive	39	86.7	9	23.1	48	57.1
	Negative	6	13.3	30	76.9	36	42.9
Total		45		39		84	100.0

Supplementary Table 3c. Crosstable comparing the conventional (paper) Amsler grid with the Digital Amsler Grid. "Positive" means the patient saw a blurry dot or missing boxes on the Amsler grid (question 1 and 3).
